# A study of the association between single nucleotide polymorphisms of the endoplasmic reticulum aminopeptidase 2 (ERAP2) gene and the risk of ankylosing spondylitis in Egyptians

**DOI:** 10.1007/s11033-024-09404-w

**Published:** 2024-05-05

**Authors:** Randa Mohamed Ibrahim Mesahel, Dina Salem Fotoh, Mahmoud Mohammed Hadhoud, Mohamed Farag Ali Assar

**Affiliations:** 1 Chemistry Department, Faculty of Science, Suze Canal University, Ismailia, Egypt; 2 Physical medicine, Rheumatology and Rehabilitation department, Faculty of Medicine, Menoufia University, Al Minufiyah, Egypt; 3 Orthopedic Surgery Department, Faculty of Medicine, Menoufia University, Al Minufiyah, Egypt; 4 Biochemistry and Molecular Biology Department, Faculty of Science, Menoufia University, Al Minufiyah, Egypt

**Keywords:** Single nucleotide polymorphisms, Endoplasmic reticulum aminopeptidase 2 (ERAP2) gene, Ankylosing spondylitis

## Abstract

**Background:**

Ankylosing spondylitis (AS) is often regarded as the prototypical manifestation of spondylo-arthropathies that prevalently involves the axial skeleton with the potential attribution of ERAP2 polymorphisms to AS predisposition. The purpose of this study was to determine the genetic association between ERAP2 gene rs2910686, and rs2248374 single nucleotide polymorphisms (SNPs) and the risk of ankylosing spondylitis in the Egyptian population.

**Methods and results:**

A cross-sectional work involved 200 individuals: 100 AS individuals diagnosed based on modified New York criteria in 1984 with 100 healthy controls matched in age and gender. The study included a comprehensive evaluation of historical data, clinical examinations, and evaluation of the activity of the disease using the Bath Ankylosing Spondylitis Disease Activity Index (BASDAI). A comprehensive laboratory and radiological evaluation were conducted, accompanied by an assessment and genotyping of the ERAP2 gene variants rs2248374 and rs2910686. This genotyping was performed utilizing a real-time allelic discrimination methodology.Highly statistically substantial variations existed among the AS patients and the healthy control group regarding rs2910686 and rs2248374 alleles. There was a statistically significant difference between rs2910686 and rs2248374 regarding BASDAI, BASFI, mSASSS, ASQoL, V.A.S, E.S.R, and BASMI in the active AS group.

**Conclusions:**

ERAP2 gene SNPs have been identified as valuable diagnostic biomarkers for AS patients in the Egyptian population being a sensitive and non-invasive approach for AS diagnosis especially rs2910686. Highly statistically significant variations existed among the AS patients and the healthy control group regarding rs2910686 alleles and genotypes.Further research is recommended to explore the potential therapeutic implications of these SNPs.

## Introduction

Ankylosing spondylitis (AS) is widely recognized as the prototypical variant of spondyloarthropathies. It is a persistent autoimmune-inflammatory condition characterized by progressive arthritis that primarily affects young adults. The disease predominantly involves the axial joints including the spine and sacroiliac joints, and is often accompanied by additional manifestations outside the joints, such as psoriasis, uveitis, and inflammatory bowel diseases [1] .

Because of the lack of precise understanding of the pathophysiology, it is hypothesized that the clinical progression of AS starts with an initial phase of inflammation, subsequently leading to the development of the formation of new bones that triggers localized osteitis, erosion of cartilage, destruction of bone, and eventual ankylosis. Sacroiliitis and syndesmophytes, known as radiographic AS, may be identified with the use of conventional radiography. However, the early diagnosis of these conditions can be facilitated by using MRI [2].

The human leukocyte antigen (HLA-B27) remains the most effective biomarker for diagnosing AS, whereas C-reactive protein (CRP) serves as the optimal marker for evaluating the extent of the disease, assessing therapy effectiveness, and monitoring structural development. However, it should be noted that HLAB-27 accounts for just 30% of the genetic variables associated with AS, suggesting the presence of other genetic abnormalities implicated in the development of AS [3].

The enzyme known as endoplasmic reticulum aminopeptidase 2 (ERAP2) is situated inside the endoplasmic reticulum. It is classified as a member of the zinc-containing metallopeptidase family, and its corresponding gene is positioned on chromosome 5q15. The involvement of ERAP2 in the process of peptide trimming via the major histocompatibility complex class I throughout the antigen presentation pathway has been observed. In contrast to ERAP1, there is a limitation of evidence on the association between ERAP2 polymorphisms and AS susceptibility [4]. Several genetic variants within the ERAP2 gene have been associated with modifications in the structure and functionality of the protein. The SNP rs2248374 in the ERAP2 gene has been shown to have a protective effect against AS. This SNP specifically affects the splicing location in exon 10 of the gene, resulting in the production of a longer exon 10 transcript [5].

ERAP2 instead has evolved under balancing selection that maintains two haplotypes, one of which undergoes RNA splicing leading to nonsense-mediated decay and loss of protein. Hence, likewise in rodents, wherein the ERAP2 gene is missing, about a quarter of the human population does not express ERAP2 [6].

The rs2248374 SNP has been previously associated with a reduced risk of AS. This SNP is a functionally significant variation located in the ERAP2 gene, that plays a crucial role in modifying the functional velocity and specificity of ERAP2 functioning during the process of trimming peptides. The rs2910686 SNP located inside the ERAP2 gene has been identified as a gene of functional significance, and its association with AS susceptibility has been established. The association between SNPs in the ERAP1 gene and susceptibility to AS has been reported [7].

The ERAP2 gene variant rs2549782 SNP exhibits linkage disequilibrium (LD) with many other ERAP2 SNPs, such as rs2548538, rs2287988, rs1056893, and rs2248374. These SNPs serve as marker variants that form haplotypes A and B, which are correlated with the protein expression of ERAP2. Furthermore, the ERAP2 gene variant rs17408150 results in a nucleotide substitution from T to A at codon 669, leading to the amino acid change from leucine to glutamine (p.Leu-669Gln). This alteration has been seen to have a significant impact on the functionality of the ERAP2 enzyme [5].

The objective of this work is to investigate the potential genetic correlation between two SNPs, namely rs2910686 and rs2248374, in the ERAP2 gene and the susceptibility to AS within Egyptian patients with AS. To the best of our knowledge, this is the first attempt to explore this link in this specific group. Additionally, an investigation was conducted to explore the potential involvement of these SNPs in regulating inflammatory markers.

## Patients and methods

Based on a previous recent study (Ebrazeh et al., 2021), found that in the instance of rs17408150, the C allele had a substantial association with a greater risk of AS in the HLA-B27- positive individuals (OR1.39,95% CI1.06-1.81, *P* = 0.013). The last sample size calculated utilizing statistics and sample size version 6 is 200 subjects, divided into two equal groups. The power of the work is 80% and the confidence level is 95% [8].

This cross-sectional work involved 200 individuals: 100 AS individuals diagnosed based on modified New York criteria in 1984 [9] recruited from the rheumatology and clinical immunology outpatient clinic, the outpatient clinic of the Rheumatology, Physical Medicine, and Rehabilitation Department, in collaboration with the Biochemistry and Molecular Biology Department, in the duration between January 2022 and July 2022 with 100 healthy controls matched in sex and age.

The present work received approval from the institutional research ethics council at our university, with the assigned IRB number 1/2022INTPH6-1. A comprehensive informed consent was collected from all participants included in the present study.

Individuals with various autoimmune illnesses, active infection, lymphoproliferative disorders, cancer, myocardial infarction, heart failure, ischemic injury, and thrombus formation, as well as patients with diabetic nephropathy, were not included in this study.

Demographic information was collected for all participants. The length of disease, specific behaviors, including smoking, clinical evaluation, and past therapeutic interventions were obtained from individuals diagnosed with AS. The assessment of the quality of life was conducted utilizing the AS quality-of-life questionnaire (ASQoL) [10], in addition to pain measurement recorded on a 10 cm visual analog scale (VAS) [11].

The evaluation of the disease’s activity was conducted by using the BASDAI, in conjunction with measurements of CRP levels in (mg/l) and ESR in mm/h [12]. The assessment of mobility and functional constraints was conducted using the Bath AS Metrology Index (BASMI) [13] and the Bath Ankylosing Spondylitis Functional Index (BASFI), respectively [14].

The participants were categorized into two distinct groups using the criteria of ESR, CRP, and BASDAI. The first group consisted of active AS individuals who exhibited a CRP level of more than 8 mg/l and/or a BASDAI score of 4 or higher, together with an ESR above 20 mm/h while the remainder of the patients were categorized as inactive AS instances.

Comprehensive laboratory analyses were conducted, which included genetic variables such as HLA-B27, antigens, inflammatory indicators (CRP and ESR) measured by the Westergren technique, and 25-hydroxyvitamin D [25(OH) D] concentrations assessed via the enzyme immunoassay method utilizing a homogeneous enzyme linked to vitamin-D binding protein.

Lateral radiographs were conducted on the cervical and lumbar spines, and the evaluation of structural harm was performed using the modified Stoke Ankylosing Spondylitis Spinal Score (mSASSS). This scoring system involves assessing the existence or lack of syndesmophytes, along the anterior margins of the lower border of C2 to the upper border of Th1, as well as from the lower border of Th12 to the upper border of S1. Each syndesmophyte is assigned a grade ranging from 0 to 3 points, with 0 indicating normal findings, 1 indicating erosion, sclerosis, or squaring, 2 indicating the existence of a syndesmophyte, and 3 indicating the formation of a bone bridge. The total score ranges from 0 to 72 [15].

Blood specimens: 6 ml of blood samples were taken under strict aseptic conditions from each person using clean venipuncture. Each sample was divided into three tubes as follows: Tube 1: EDTA-contained sterile tube kept at -20 C for further DNA extraction for HLAB27 analysis and genotyping of rs2248374 and rs2910686 of ERAP2 gene, Tube 2: Sodium citrate sterile tube presents at room temperature for measurement of ESR, Tube 3: sterile plain tube that allowed to clot at 37° C, and used for measurement of CRP and vitamin D after serum separation by centrifugation.

DNA was extracted from whole blood by a GeneJET Genomic DNA Purification Kit (Thermo Scientific, Lithuania. cat# K0721) following the manufacturer’s protocol. DNA concentration, quality, and purity were assessed using a Nanophotometer N-60 (Implen, Germany).

HLAB27 analysis was done using the HLA-B27 REAL-TIME PCR Genotyping Kit with a PCR mix that includes the internal control (IC B27). ICB 27 serves as sample intake control and allows to evaluation of the quantity of genomic DNA. It is needed for assurance of PCR quality and sufficiency of input DNA. The interpretation of the result for each sample is carried out automatically concerning the Threshold cycle (CT) for Fam (specific dye label) and Hex (internal control dye label) channels.

**Fig. 1:**
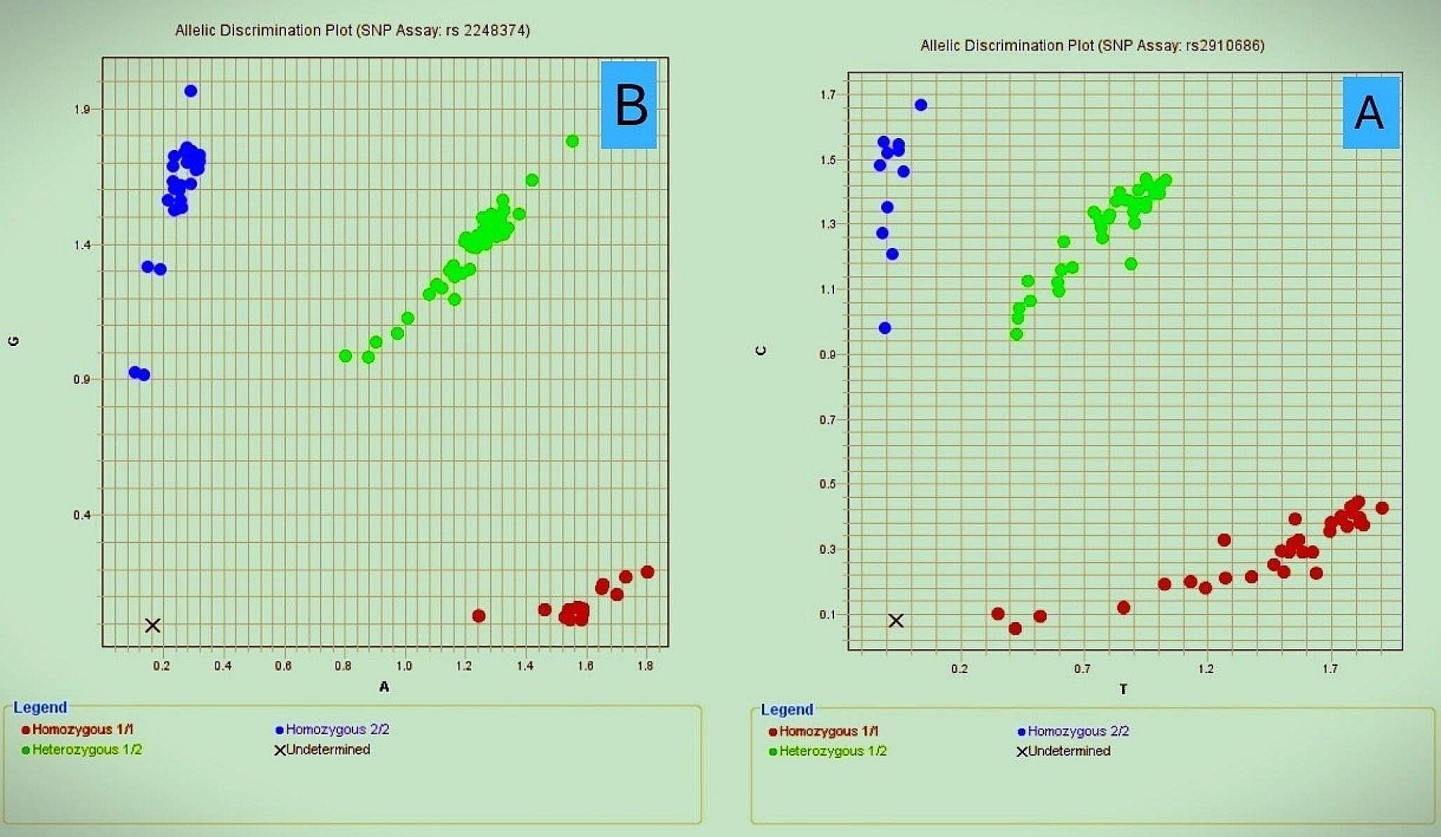
**A**: Allelic discrimination plot (SNP Assay: rs2910686), **B**: Allelic discrimination plot (SNP Assay: rs2248374)

ERAP2 gene rs2248374 and rs2910686 SNPs genotyping was done using an allelic discrimination assay by real-time PCR using a TaqMan probe (Applied Biosystems, Foster City, USA) with TaqMan SNP Genotyping Assays. Master Mix II (2x), primers, and probes were supplied by Thermo Fisher Scientific and Catalog number (Catalog No D3024). (Fig. 1a and b, respectively)

### Detection of ERAP2 gene rs2248374 and rs2910686 SNPs

Thermal cycling conditions: Following the PCR amplification process, the endpoint plate was subjected to analysis utilizing the Applied Biosystems Real-Time PCR System. The Sequence Detection System Software utilizes the fluorescence measures obtained throughout the plate read to provide a graphical representation of fluorescence (Rn) values, which are derived from the signals detected in each well. The fluorescence signals that have been displayed provide information on the presence of certain alleles in all samples.

## Statistical analysis of the data

The data were inputted into the computer and then analyzed utilizing the IBM SPSS software program, version 20.0. (IBM Corp, Armonk, NY). The representation of categorical data included the use of numerical values and percentages. The chi-square test was used to conduct a comparative analysis among two distinct groups. In cases where the predicted count of < 5 was seen in over 20% of the cells, the Fisher Exact or Monte Carlo adjustment test was used as an alternative method. The normality of the continuous data was assessed using the Kolmogorov-Smirnov test. The quantitative data were presented in terms of the range (minimum and maximum values), mean, standard deviation, and median for parameters that followed a normal distribution. The F-test, (ANOVA), has been employed to assess the differences across many groups. Additionally, a Post Hoc test, specifically the Tukey test, was conducted to do pairwise comparisons among the groups. In contrast, when dealing with quantitative parameters that do not follow a normal distribution, the Whitney test was employed for contrasting two groups, whilst the Kruskal-Wallis test was utilized to contrast more than two groups under study. Additionally, pairwise comparisons were conducted using the Post Hoc method, specifically Dunn’s multiple comparisons test. The significance of the obtained results was judged at the 5% level.

## Results

A total of one hundred individuals diagnosed with AS were categorized into two groups, namely active and inactive, using criteria such as ESR, CRP, and BASDAI. A similar number of controls, matched in terms of age and sex, also participated in the study.

### Demographic and laboratory data of patients and healthy controls

There were significant differences among active ankylosing spondylitis (group I) and healthy control (group III) regarding smoking habit (*P* < 0.001). additionally, a significant increase was found in active ankylosing spondylitis, inactive ankylosing spondylitis (group II), and healthy control regarding ESR, CRP, and vitamin D levels, but there were non-significant differences between active ankylosing spondylitis, inactive ankylosing spondylitis and healthy control regarding age, sex, occupation, disease duration, and drug taking (Table 1).

**Table 1:** Comparison between the three studied groups according to demographic data and clinical data

	Group I (*n* = 50)	Group II (*n* = 50)	Group III (*n* = 100)	*p*	Sig. bet. grps.		
					I vs. II	I vs. III	II vs. III
**Sex**							
Male	34 (68%)	38 (76%)	60 (60%)	^χ2^*p*=0.141	> 0.05	> 0.05	> 0.05
Female	16 (32%)	12 (24%)	40 (40%)				
**Occupation**							
Working	32 (64%)	35 (70%)	66 (66%)	^χ2^*p*=0.808	> 0.05	> 0.05	> 0.05
Not working	18 (36%)	15 (30%)	34 (34%)				
**Age (years)**							
Mean ± SD.	35.16 ± 7.82	37.48 ± 10.35	38.27 ± 9.80	^F^*p*=0.168	> 0.05	> 0.05	> 0.05
Median (Min.–Max.)	35 (22–52)	35 (22–60)	36(22–64)				
**Smoking**							
Smoker	24 (48%)	15 (30%)	20 (20%)	^χ2^*p*=0.002	0.065	< 0.001	0.172
Non-smoker	26 (52%)	35 (70%)	80 (80%)				
**Disease duration(years)**							
Mean ± SD.	10.18 ± 4.93	9.02 ± 5.53	–	^U^*p*=0.167	–	–	–
Median (Min.–Max.)	9.50 (2–25)	8.50 (2–20)	–				
**Drugs**							
No	7 (14.0%)	2 (4.0%)	–	^χ2FE^*p*=0.160	–	–	–
Yes	43 (86.0%)	48 (96.0%)	–				
NSAID	43 (86.0%)	48 (96.0%)	–	^χ2FE^=0.160	–	–	–
Sulfasalazine	11 (22.0%)	12 (24.0%)	–	^χ2^*p*=0.812	–	–	–
MTX	11 (22.0%)	5 (10.0%)	–	^χ2^*p*=0.102	–	–	–
**E.S. R**							
Mean ± SD.	35.18 ± 6.57	10.44 ± 4.40	3.78 ± 0.68	^H^*p*<0.001	< 0.001	< 0.001	< 0.001
Median (Min.–Max.)	35.5(24–47)	9.50 (5–19)	3.8(2.4–4.9)				
**C.R. P**							
Mean ± SD.	50.36 ± 8.53	6.79 ± 0.72	3.38 ± 0.93	^H^*p*<0.001	< 0.001	< 0.001	< 0.001
Median (Min.–Max.)	52.0 (35–66)	6.85 (5.5–8)	3.45 (2–5)				
**Vitamin D**							
Mean ± SD.	16.05 ± 2.73	23.45 ± 1.83	36.60 ± 6.40	^H^*p*<0.001	< 0.001	< 0.001	< 0.001
Median (Min.–Max.)	16.5(9.8–20.3)	23.3(20.9–28.8)	34.7(29–50)				

SD: Standard deviation U: Mann Whitney test F: F for One way ANOVA test

χ^2^: Chi square test FE: Fisher Exact

H: H for Kruskal Wallis test, pairwise comparison bet. each 2 groups were done using Post Hoc Test (Dunn’s for multiple comparisons test)

*p*: *p* value for comparing between the three studied groups

^HW^p1: *p* value for Chi square for goodness of fit for Hardy-Weinberg equilibrium

*: Statistically significant at *p* ≤ 0.05

### Univariate regression analysis for alleles of rs2248374 and rs2910686 between cases and controls

rs2248374 SNP G allele was more frequent in the AS patient group (54%) compared to the healthy control group (49.5%), but the difference was not statistically significan. The frequency of the rs2910686 SNP C allele was significantly higher in the AS patient group compared to the healthy control group ( 94(47%) versus 50(25%), respectively) with OR (LL – UL 95%C.I) of 2.660 (1.741–4.064) and significant *p*-value (*p* < 0.001) (Table 2).

**Table 2:** Univariate regression analysis for alleles of rs2248374 and rs2910686 between cases and controls

	Group I + II (*n* = 100)	Group III (*n* = 100)	^χ2^p	OR (LL – UL 95% C. I)	*p*_2_
**rs2248374**					
AA	20(20%)	27 (27.0%)	0.504		
AG	52(52%	47 (47.0%)		1.494(0.742–3.008)	0.261
GG	28(28%)	26 (26.0%)		1.454(0.662–3.194)	0.351
^**HW**^**p**_**1**_	**0.640**	**0.549**			
**Allele**					
A	92(46%)	101 (50.5%)	0.368		
G	108(54%)	99 (49.5%)		1.198(0.809–1.774)	0.368
**rs2910686**					
TT	28(28%)	58(58%)	< 0.001^*^		
TC	50(50%)	34(34%)		3.046(1.627–5.703)	< 0.001^*^
CC	22(22%	8(8%)		5.696(2.256–14.386)	< 0.001^*^
^**HW**^**p**_**1**_	**0.971**	**0.351**			
**Allele**					
T	106(53%)	150(75%)	< 0.001^*^		
C	94(47%)	50(25%)		2.660(1.741–4.064)	< 0.001^*^

χ^2^: Chi square test

*p*: *p* value for comparing between the three studied groups

^HW^p1: *p* value for Chi square for goodness of fit for Hardy-Weinberg equilibrium

p_2_: *p* value for Univariate regression analysis for comparing with the reference genotype

Significant differences were also observed for the frequencies of the genotypes for this SNP between AS cases and controls. The number of carriers of the minor allele C (genotypes TC and CC) was significantly higher in patients compared to controls (72% versus 42%, *P* < 0.001) (Table 2).

### Activity indices, clinical and radiological data of the two patient groups

A statistically significant increase was observed in BASDAI (*P* < 0.001), BASFI (*P* = 0.001), BASMI (*P* = 0.005), mSASSS(*P* < 0.001), ASQoL(*P* < 0.001), V.A.S(*P* < 0.001) in individuals with active ankylosing spondylitis when contrasted to those with inactive ankylosing spondylitis. Also, there is an increased percentage of patients with clinical symptoms in the form of Knee arthritis, Enthesitis, and Uveitis with an x-ray indicating Ankylosed cervical spine in individuals with active ankylosing spondylitis when contrasted to inactive ankylosing spondylitis (Table 3).

**Table 3:** Comparison between the two studied groups according to different parameters

	Group I (*n* = 50)	Group II (*n* = 50)		*p*
**BASDAI**				
Mean ± SD.	5.11 ± 0.73	2.68 ± 0.73		< 0.001
Median (Min.–Max.)	5.15 (3.9–6.4)	2.75 (1.3–3.8)		
**BASFI**				
Mean ± SD.	4.86 ± 0.99	2.50 ± 0.66		< 0.001
Median (Min.–Max.)	4.75 (3.4–6.8)	2.65 (1–3.4)		
**BASMI**				
Mean ± SD.	4.59 ± 0.51	2.46 ± 0.59		< 0.001
Median (Min.–Max.)	4.65 (3.5–5.3)	2.55 (1.4–3.30)		
**mSASSS**				
Mean ± SD.	31.25 ± 1.94	17.71 ± 1.98		< 0.001
Median (Min.–Max.)	31.43 (27.5–34.6)	17.93 (13.6–20.5)		
**ASQoL**				
Mean ± SD.	7.90–14.30	1.0–7.80		< 0.001
Median (Min.–Max.)	9.90 (7.9–14.3)	6.65 (1–7.8)		
**V.A. S**				
Mean ± SD.	7.04 ± 1.28	4.12 ± 0.90		< 0.001
Median (Min.–Max.)	7.0 (5–9)	4.0 (2–5)		
**HLAB27**				
Negative	17 (34%)	17 (34%)		1.000
Positive	33 (66%)	33 (66%)		
**Clinical symptoms**				
No	23 (46%)	7 (14%)		< 0.001
Yes	27 (54%)	43 (86%)		
Knee arthritis	6 (12%)	0 (0%)		^FE^*p*=0.027
Enthesitis	17 (34%)	41 (82%)		< 0.001
Uveitis	10 (20%)	3 (6%)		0.037
**X-ray**				
Bil Sacroiliitis	44 (88.0%)	42 (84.0%)		0.564
Bamboo spine	17 (34.0%)	11 (22.0%)		0.181
Ankylosed cervical spine	8 (16.0%)	0 (0.0%)		^FE^*p*=0.006
RT sacroiliitis	6 (12.0%)	3 (6.0%)		0.487

SD: Standard deviation U: Mann Whitney test

χ^2^: Chi square test FE: Fisher Exact

p: *p* value for comparing between the two studied groups

*: Statistically significant at *p* ≤ 0.05

### Relation between (rs2248374) gene polymorphism and demographic, laboratory data, and clinical data of patients with active ankylosing spondylitis

A statistically significant difference of greater BASDAI(*P* < 0.001), BASFI(*P* = 0.001), BASMI(*P* = 0.005), mSASSS (*P* < 0.001), ASQoL(*P* < 0.001), V.A.S(*P* < 0.001), E.S.R (*P* = 0.001)and C.R.P (*P* < 0.001), was observed in GG compared to the other genotypes (Table 4).

**Table 4:** Relation between rs2248374 and different parameters in group I

	rs2248374				*p*
	AA (*n* = 8)	AG (*n* = 21)	GG (*n* = 21)		
**Sex**					
Male	7 (87.5%)	12 (57.1%)	15 (71.4%)		0.266
Female	1 (12.5%)	9 (42.9%)	6 (28.6%)		
**Occupation**					
Working	7 (87.5%)	8 (38.1%)	17 (81.0%)		0.005
Not working	1 (12.5%)	13 (61.9%)	4 (19.0%)		
**Age (years)**	38.9 ± 9.88	34.4 ± 8.03	34.5 ± 6.68		0.348
**Smoking**					
Smoker	7 (87.5%)	6 (28.6%)	11 (52.4%)		^MC^p= 0.015
Non-smoker	1 (12.5%)	15 (71.4%)	10 (47.6%)		
**Disease duration (years)**	7 (5–25)	10 (2–19)	8 (2–17)		0.494
**Drugs**					
No	2 (25.0%)	5 (23.8%)	0 (0.0%)		^MC^*p*=0.037
**Yes**	6 (75.0%)	16 (76.2%)	21 (100.0%)		
NSAID	6 (75.0%)	16 (76.2%)	21 (100.0%)		^MC^*p*=0.037
Sulfasalazine	1 (12.5%)	5 (23.8%)	5 (23.8%)		^MC^*p*=0.821
MTX	2 (25.0%)	6 (28.6%)	3 (14.3%)		^MC^*p*=0.582
**BASDAI**	4.60 (3.90–6)	5 (4–6.30)	5.60 (3.90–6.40)		< 0.001
**BASFI**	4 (3.50–5.90)	4.40 (3.50–6.60)	5.60 (3.40–6.80)		0.001
**BASMI**	4.20 (3.50–5.30)	4.50 (3.70–5.30)	5 (3.50–5.30)		0.005
**mSASSS**	30.5 (27.5–33.2)	31.2 (27.6–34.5)	31.9 (27.5–34.6)		< 0.001
**ASQoL**	8.80 (7.90–12.2)	9.40 (8–14)	10.9 (7.90–14.3)		< 0.001
**V.A. S**	6 (5–9)	7 (5–9)	8 (5–9)		< 0.001
**E.S. R**	31 (24–43)	34 (25–45)	40 (24–47)		0.001
**C.R. P**	44 (35–60)	50 (38–64)	56 (35–66)		< 0.001
**Vitamin D**	17.7 (12.4–20.3)	16.8 (9.90–19.8)	15.4 (9.80–20.3)		0.182
**HLAB27**					
Negative	4 (50.0%)	9 (42.9%)	4 (19.0%)		0.154
Positive	4 (50.0%)	12 (57.1%)	17 (81.0%)		
**Clinical symptoms**					
No	5 (62.5%)	10 (47.6%)	8 (38.1%)		^MC^*p*=0.557
**Yes**					
Knee arthritis	13 (61.9%)	11 (52.4%)	3 (37.5%)		^MC^*p*=0.373
Enthesitis	4 (19.0%)	1 (12.5%)	1 (4.8%)		0.788
Uveitis	8 (38.1%)	6 (28.6%)	3 (37.5%)		^MC^*p*=0.363
**X-ray**					
Bil Sacroiliitis	8 (100.0%)	16 (76.2%)	20 (95.2%)		^MC^*p*=0.144
Bamboo spine	3 (37.5%)	7 (33.3%)	7 (33.3%)		0.974
Ankylosed cervical spine	0 (0.0%)	2 (9.5%)	6 (28.6%)		^MC^*p*=0.142
RT sacroiliitis	0 (0.0%)	5 (23.8%)	1 (4.8%)		^MC^*p*=0.145

SD: Standard deviation F: F for One way ANOVA test H: H for Kruskal Wallis test

χ^2^: Chi square test MC: Monte Carlo

p: *p* value for Relation between rs2248374 and different parameters

*: Statistically significant at *p* ≤ 0.05

### Relation between (rs2910686) gene polymorphism and demographic, laboratory data, and clinical data of patients with active ankylosing spondylitis

A statistically significant difference of greater BASDAI(*P* < 0.001), BASFI(*P* < 0.001), BASMI(*P* = 0.009), mSASSS(*P* < 0.001), ASQoL(*P* < 0.001), V.A.S(*P* < 0.001), E.S.R(*P* < 0.001), C.R.P (*P* < 0.001) and vitamin D (*P* = 0.01), was existed in TT genotypes contrasted to the other two genotypes (Table 5).

**Table 5:** Relation between rs2910686 and different parameters in group I

	rs2910686				*p*
	TT (*n* = 14)	TC (*n* = 23)	CC (*n* = 13)		
**Sex**					
Male	11 (78.6%)	15 (65.2%)	8 (61.5%)		^MC^*p*=0.637
Female	3 (21.4%)	8 (34.8%)	5 (38.5%)		
**Occupation**					
Working	12 (85.7%)	12 (52.2%)	8 (61.5%)		0.117
Not working	2 (14.3%)	11 (47.8%)	5 (38.5%)		
**Age (years)**	34.86 ± 7.18	35.91 ± 5.70	34.15 ± 11.50		0.805
**Smoking**					
Smoker	7 (50%)	9 (39.1%)	8 (61.5%)		0.427
Non-smoker	7 (50%)	14 (60.9%)	5 (38.5%)		
**Disease duration (years)**	9.50 (2–17)	10 (2–19)	6 (2–25)		0.291
**Drugs**					
No	0 (0.0%)	2 (8.7%)	5 (38.5%)		^MC^*p*=0.014
**Yes**	**14 (100.0%)**	**21 (91.3%)**	**8 (61.5%)**		
NSAID	14 (100.0%)	21 (91.3%)	8 (61.5%)		^MC^*p*=0.014
Sulfasalazine	3 (21.4%)	5 (21.7%)	3 (23.1%)		^MC^*p*=1.000
MTX	3 (21.4%)	7 (30.4%)	1 (7.7%)		^MC^*p*=0.340
**BASDAI**	5.30 (4.10–6.40)	5.20 (4–6.30)	4.70 (3.90–6)		< 0.001
**BASFI**	5 (3.40–6.80)	4.90 (3.50–6.60)	4.20 (3.50–5.90)		< 0.001
**BASMI**	4.90 (3.80–5.30)	4.50 (3.70–5.10)	4.50 (3.50–5.30)		0.009
**mSASSS**	31.8 (27.7–34.6)	31.6 (27.6–34.5)	30.5 (27.5–33.2)		< 0.001
**ASQoL**	10.2 (8.10–14.3)	10 (8.0–14.0)	8.90 (7.90–12.20)		< 0.001
**V.A. S**	7.50 (5–9.0)	7 (5.0–9.0)	6 (5–9)		< 0.001
**E.S. R**	37 (25–47)	36 (25–45)	32 (24–43)		< 0.001
**C.R. P**	53 (39–66)	52 (38–64)	45 (35–60)		< 0.001
**Vitamin D**	16.0 (9.80–19.70)	16.3 (9.90–19.80)	17.5 (12.4–20.3)		0.010
**HLAB27**					
Negative	2 (14.3%)	10 (43.5%)	5 (38.5%)		^MC^*p*=0.182
Positive	12 (85.7%)	13 (56.5%)	8 (61.5%)		
**Clinical symptoms**					
No	6 (42.9%)	12 (52.2%)	5 (38.5%)		^MC^*p*=0.703
**Yes**	8 (57.1%)	11 (47.8%)	8 (61.5%)		
Knee arthritis	1 (7.1%)	3 (13.0%)	2 (15.4%)		^MC^*p*=0.871
Enthesitis	5 (35.7%)	7 (30.4%)	5 (38.5%)		^MC^*p*=0.929
Uveitis	3 (21.4%)	4 (17.4%)	3 (23.1%)		^MC^*p*=0.905
**X-ray**					
Bil Sacroiliitis	13 (92.9%)	21 (91.3%)	10 (76.9%)		^MC^*p*=0.455
Bamboo spine	5 (35.7%)	10 (43.5%)	2 (15.4%)		^MC^*p*=0.280
Ankylosed cervical spine	3 (21.4%)	4 (17.4%)	1 (7.7%)		^MC^*p*=0.705
RT sacroiliitis	1 (7.1%)	2 (8.7%)	3 (23.1%)		^MC^*p*=0.461

SD: Standard deviation F: F for One way ANOVA test H: H for Kruskal Wallis test

χ^2^: Chi square test MC: Monte Carlo

p: *p* value for Relation between rs2910686 and different parameters

*: Statistically significant at *p* ≤ 0.05

### Relation between (rs2248374) gene polymorphism and demographic, laboratory data, and clinical data of patients with inactive ankylosing spondylitis

There were no statistically significant differences among different parameters and the three types of genotypes (Table 6).

**Table 6:** Relation between rs2248374 and different parameters in group II

	rs2248374				*p*
	AA (*n* = 12)	AG (*n* = 31)	GG (*n* = 7)		
**Sex**					
Male	9 (75.0%)	24 (77.4%)	5 (71.4%)		^MC^p= 1.000
Female	3 (25.0%)	7 (22.6%)	2 (28.6%)		
**Occupation**					
Working	7 (58.3%)	23 (74.2%)	5 (71.4%)		^MC^p= 0.616
Not working	5 (41.7%)	8 (25.8%)	2 (28.6%)		
**Age (years)**					
Mean ± SD.	37.33 ± 12.06	37.77 ± 9.75	36.43 ± 11.44		0.953
**Smoking**					
Smoker	1 (8.3%)	14 (45.2%)	0 (0.0%)		^MC^p= 0.012
Non-smoker	11 (91.7%)	17 (54.8%)	7 (100.0%)		
**Disease duration (years)**	7.50 (3–20)	9 (2–20)	8 (2–20)		0.845
**Drugs**					
No	1 (8.3%)	1 (3.2%)	0 (0.0%)		^MC^p= 0.620
**Yes**	11 (91.7%)	30 (96.8%)	7 (100.0%)		
NSAID	11 (91.7%)	29 (93.5%)	6 (85.7%)		^MC^*p*=0.767
Sulfasalazine	1 (8.3%)	8 (25.8%)	3 (42.9%)		^MC^*p*=0.222
MTX	0 (0.0%)	4 (12.9%)	1 (14.3%)		^MC^*p*=0.503
**BASDAI**	2.65 (1.70–3.60)	2.80 (1.30–3.80)	2.70 (1.30–3.30)		0.715
**BASFI**	2.55 (1.60–3.20)	2.70 (1.0–3.40)	2.60 (1.0–3.11)		0.836
**BASMI**	2.45 (1.60–3.10)	2.60 (1.40–3.30)	2.50 (1.40–3.10)		0.936
**mSASSS**	17.8 (14.8–19.8)	18.7 (13.6–20.5)	17.2 (13.6–19.4)		0.706
**ASQoL**	6.55 (1.90–7.50)	6.70 (1–7.80)	6.60 (1–7.20)		0.715
**V.A. S**	4 (3–5)	4 (2–5)	4 (2–5)		0.666
**E.S. R**	9.0 (5.0–17.0)	10.0 (5.0–19.0)	9.0 (5.0–14.0)		0.728
**C.R. P**	6.75 (5.80–7.70)	6.90 (5.50–8.0)	6.80 (5.50–7.40)		0.715
**Vitamin D**	23.6 (21.5–25.3)	23.2 (20.9–28.8)	23.3 (21.8–28.8)		0.715
**HLAB27**					
Negative	3 (25.0%)	13 (41.9%)	1 (14.3%)		^MC^p= 0.332
Positive	9 (75.0%)	18 (58.1%)	6 (85.7%)		
**Clinical symptoms**					
No	1 (8.3%)	5 (16.1%)	1 (14.3%)		^MC^p= 0.852
**Yes**	11 (91.7%)	26 (83.9%)	6 (85.7%)		
Knee arthritis	0 (0.0%)	0 (0.0%)	0 (0.0%)		–
Enthesitis	11 (91.7%)	24 (77.4%)	6 (85.7%)		^MC^*p*=0.668
Uveitis	0 (0.0%)	3 (9.7%)	0 (0.0%)		^MC^*p*=0.713
**X-ray**					
Bil. sacroiliitis	10 (83.3%)	26 (83.9%)	6 (85.7%)		^MC^*p*=1.000 ^MC^*p*=0.607 – ^MC^*p*=0.483
Bamboo spine	4 (33.3%)	6 (19.4%)	1 (14.3%)		
Ankylosed cervical spine	0 (0.0%)	0 (0.0%)	0 (0.0%)		
RT sacroiliitis	0 (0.0%)	2 (6.5%)	1 (14.3%)		

SD: Standard deviation F: F for One way ANOVA test H: H for Kruskal Wallis test

χ^2^: Chi square test MC: Monte Carlo

p: *p* value for Relation between rs2248374 and different parameters

*: Statistically significant at *p* ≤ 0.05

### Relation between (rs2910686) gene polymorphism and demographic, laboratory data, and clinical data of patients with inactive ankylosing spondylitis

A significant difference of greater BASDAI(*P* = 0.013), BASFI(*P* = 0.011), BASMI(*P* = 0.012), mSASSS(*P* = 0.013), E.S.R(*P* = 0.027), C.R.P(*P* = 0.027) and lower vitamin D (*P* = 0.027), was observed in TT genotype contrasted to the other two genotypes (Table 7).

**Table 7:** Relation between rs2910686 and different parameters in group II

	rs2910686				*p*
	TT (*n* = 14)	TC (*n* = 27)	CC (*n* = 9)		
**Sex**					
Male	11 (78.6%)	21 (77.8%)	6 (66.7%)		^MC^p= 0.820
Female	3 (21.4%)	6 (22.2%)	3 (33.3%)		
**Occupation**					
Working	10 (71.4%)	19 (70.4%)	6 (66.7%)		^MC^p= 1.000
Not working	4 (28.6%)	8 (29.6%)	3 (33.3%)		
**Age (years)**	35.29 ± 8.77	38.70 ± 9.26	37.22 ± 15.44		0.612
**Smoking**					
Smoker	1 (7.1%)	12 (44.4%)	2 (22.2%)		^MC^p= 0.049
Non-smoker	13 (92.9%)	15 (55.6%)	7 (77.8%)		
**Disease duration (years)**	9 (3–20)	9 (2.50–20)	7 (2–12)		0.546
**Drugs**					
No	0 (0.0%)	1 (3.7%)	1 (11.1%)		^MC^p= 0.405
**Yes**	14 (100.0%)	26 (96.3%)	8 (88.9%)		
NSAID	13 (92.9%)	25 (92.6%)	8 (88.9%)		^MC^*p*=1.000
Sulfasalazine	6 (42.9%)	6 (22.2%)	0 (0.0%)		^MC^*p*=0.069
MTX	1 (7.1%)	4 (14.8%)	0 (0.0%)		^MC^*p*=0.569
**BASDAI**	3.35 (1.80–3.80)	2.60 (1.50–3.60)	2.50 (1.30–3.20)		0.013
**BASFI**	3.11 (1.70–3.40)	2.50 (1.40–3.20)	2.40 (1.00–3.00)		0.011
**BASMI**	3.05 (1.70–3.30)	2.40 (1.40–3.10)	2.30 (1.40–3.00)		0.012
**mSASSS**	19.5 (15.4–20.5)	17.1 (13.9–19.8)	16.95 (13.6–19.3)		0.013
**ASQoL**	7.20 (1.90–7.80)	6.50 (1.70–7.50)	6.40 (1–7.10)		0.085
**V.A. S**	5 (3–5)	4 (3–5)	4 (2–5)		0.195
**E.S. R**	14.0 (6.0–19.0)	9.0 (5.0–17.0)	8 (5–13)		0.027
**C.R. P**	7.40 (5.90–8.0)	6.70 (5.60–7.70)	6.60 (5.50–7.30)		0.027
**Vitamin D**	21.8 (20.9–25.2)	23.9 (21.5–25.5)	24 (21.9–28.80)		0.027
**HLAB27**					
Negative	6 (42.9%)	9 (33.3%)	2 (22.2%)		^MC^p= 0.619
Positive	8 (57.1%)	18 (66.7%)	7 (77.8%)		
**Clinical symptoms**					
No	2 (14.3%)	4 (14.8%)	1 (11.1%)		^MC^p= 1.000
**Yes**	12 (85.7%)	23 (85.2%)	8 (88.9%)		
Knee arthritis	0 (0.0%)	0 (0.0%)	0 (0.0%)		–
Enthesitis	11 (78.6%)	22 (81.5%)	8 (88.9%)		^MC^*p*=1.000
Uveitis	1 (7.1%)	2 (7.4%)	0 (0.0%)		^MC^*p*=1.000
**X-ray**					
Bil Sacroiliitis	14 (100.0%)	21 (77.8%)	7 (77.8%)		^MC^*p*=0.119
Bamboo spine	4 (28.6%)	6 (22.2%)	1 (11.1%)		^MC^*p*=0.668
Ankylosed cervical spine	0 (0.0%)	0 (0.0%)	0 (0.0%)		–
RT sacroiliitis	0 (0.0%)	1 (3.7%)	2 (22.2%)		^MC^*p*=0.138

SD: Standard deviation F: F for One way ANOVA test H: H for Kruskal Wallis test

χ2: Chi square test MC: Monte Carlo

p: *p* value for Relation between rs2910686 and different parameters

*: Statistically significant at *p* ≤ 0.05

## Discussion

Ankylosing spondylitis (AS) is a chronic inflammatory autoimmune condition that is distinguished by the presence of pathologic new bone growth [16]. The origin, pathophysiology, and diagnosis of AS continue to provide significant challenges in the field. The HLA-B27 antigens have been widely seen in individuals with AS, making it a significant diagnostic biomarker. However, it is important to note that the contribution of this factor to the total genetic susceptibility of AS is below 30%. In addition, it is important to note that X-rays are not capable of detecting first radiological alterations, while the use of MRI as a screening tool is hindered by its high cost and impractical handling. Hence, there is a critical requirement for the identification of new biomarkers to facilitate the screening and diagnosis of AS.

The accurate diagnostic and prognostic considerations of AS remain unaddressed medical needs. The prioritization of care for individuals with AS is crucial due to the detrimental effects of this chronic inflammatory condition on young people, posing risks to their daily activities, productivity, and overall quality of life. This, in turn, leads to an enormous financial burden on a national scale [16, 17].

The primary objective of this research is to investigate the potential genetic correlation between two specific SNPs, namely rs2910686 and rs2248374, inside the ERAP2 gene, and the susceptibility to AS. To the best of our knowledge, this study represents the first attempt to explore this link in a cohort of 100 individuals with AS within our nation. Additionally, an investigation was conducted to explore the potential involvement of these SNPs in regulating inflammatory markers.

The cohort of participants with active AS had a lower mean age (35.7 ± 7.9 years) compared to those with inactive AS. Furthermore, the active AS group had a longer average duration of illness (25 ± 2.4 years) and mostly consisted of male individuals (69%). This gender distribution aligns with the typical epidemiological trend of AS, whereby men are afflicted at a frequency of 2 to 4 times higher than females. Our findings are consistent with evidence from the literature that has been published and reflects the disease’s youthful character, which makes it more aggressive in young people and has a peak age of onset between the 2nd and 3rd decades [15]. Numerous investigations have implicated the male gender and extended illness duration as unfavorable prognostic indicators for ankylosing spondylitis [18]. But also it was shown that both males and females had functional incapacities and structural damage [18].

In the present study, it was observed that there was a deficiency of vitamin D in the active and inactive groups of individuals with AS, correspondingly. Notably, there was a substantial decrease in the number of active AS instances (16.05 ± 2.73), which suggests that inadequate levels of vitamin D may play a major role in the development and progression of the illness. This statement aligns with several recent studies that have provided evidence for the involvement of vitamin D in AS via influencing both the adaptive and innate immune systems, rather than only affecting calcium metabolism. These findings suggest the potential benefits of supplemental vitamin D for individuals with this particular condition [19, 20].

There are conflicting literature results about the correlation between vitamin D and ankylosing spondylitis (AS), as shown by the research conducted by Zhao et al., [20], which refuted the notion of vitamin D having any immunomodulatory impact on AS disease activity.

HLA-B27 has traditionally served as the primary diagnostic marker for AS, despite the recognition that this class 1 human leukocyte antigen accounts for just 30% of the overall hereditary risk associated with AS [21]. The research conducted by Neves et al., revealed that HLAB-27 had a high prevalence rate of 96.8% among a cohort consisting of individuals of Caucasian descent. The prevalence of HLAB-27 in AS sufferers was found to be 66% in this research. The observed disparity might perhaps be ascribed to the racial genetic variability within the groups under investigation [19]. The findings may validate the pursuit of novel diagnostic markers for AS that provide improved accuracy.

In the present work, we revealed that there were highly statistically significant differences among the active AS patients and inactive AS patients regarding BASDAI, BASFI, BASMI, mSASSS, ASQoL, and V.A.S.

Our findings were in line with Maghraoui et al., who found that statistical disparities were seen in symptomatic severity measures, including the Schöber test, BASMI, chest expansion, BASFI, BASDI, and BASG, as well as structural severity metrics, such as lumbar syndesmophytes score and BASRI when comparing patients with active AS to patients with inactive AS [22].

In their study, Londono et al. observed statistically significant variations among the patients under investigation in terms of their scores on BASDAI and BASFI, as well as the overall assessment of the disease’s activity conducted by the physician [23].

This research provides evidence of substantial and statistically significant variations between the groups of individuals with AS and the control group in E.S.R and C.R.P. In a similar vein, Ebrazeh et al., [8] observed a statistically significant elevation (*P* < 0.0001) in the C-reactive protein level among those in the case group (3.14 ± 2.27) compared to those in the control group (1.27 ± 0.95). Furthermore, Robinson et al., [24] discovered a statistically significant elevation in levels of E.S.R and C.R.P in individuals with AS when contrasted with the control group (*p* < 0.001).

In a study conducted by Robinson et al., [24] in 2015, the authors examined specific subgroups of HLA-B27 positive AS-associated ERAP1 haplotypes, which were identified by the genetic markers rs30187 and rs10050860. The study also included HLA-B27 positive and unselected controls of European descent from the IGAS study. The researchers discovered a correlation between the ERAP2 polymorphism rs2248374 and AS in individuals who tested positive for HLA-B27. These investigations revealed a significant correlation between ERAP2 and HLA-B27-positive AS individuals.

The research conducted by Cortes et al., [25] in 2013 included a total of 10,619 patients diagnosed with AS and 15,145 control subjects of East Asian, European, and Latin American descent. The researchers identified a correlation between AS and two SNPs located in the ERAP2 gene, namely rs2549782 and rs2248374. This link was seen among individuals who tested positive for the HLA-B27 antigen. Upon doing a controlled analysis of the ERAP1 interaction with the ERAP2 polymorphism, a significant connection was identified between ERAP2 SNPs and AS instances. Specifically, the rs2910686 SNP exhibited an OR of 1.19 and a *p*-value of 0.213 × 10 − 2. The study conducted by Ebrazeh et al., [8] revealed a substantial statistical connection between the rs2910686 SNPs and the risk of AS.

In the present study, there were significant associations between ERAP2 polymorphism rs2248374, rs2910686, and risk of AS. A statistically significant difference was observed in the frequency of the genotype and allelic distribution of rs2248374 and rs2910686 SNPs among the individuals with AS and the healthy control group, with statistically significant differences (*p* values = 0.023 and < 0.001, respectively).

Wiśniewski et al., observed no effect of rs2248374 in ERAP2 gene alone on the risk of AS in Polish population.They found associations with ERAP1-ERAP2 haplotypes. The strongest association with AS was observed for rs30187 of ERAP1 gene.The minor T allele and homozygous TT genotype of this SNP significantly increased disease risk (OR = 1.56, 95%CI = 1.22–1.99, *p* = 0.0004 and OR = 2.52, 95%CI = 1.50–4.25, *p* = 0.001, respectively) [7].

Paladini et al., also observed no association of ERAP2 rs2248374 with the risk of AS in Sardinian population [26]. They found associations with SNP rs75862629.

In contrast to our findings, Su et al. found no association between SNPs of ERAP1/ERAP2/RUNX3 and susceptibility of AS with or without AAU. A case-control study between patients with human leucocyte antigen HLA-B27-positive and healthy controls also failed to demonstrate an association of the tested SNP with AS with or without AAU. Moreover, a meta-analysis showed that there was no association between rs30187, rs27037, rs27980, rs27434, and rs27582 in ERAP1 with AS in Chinese Han. Taken together, 17 SNPs in ERAP1/ERAP2 and RUNX3 genes did not confer disease susceptibility to AS in Chinese Han [27].

Gao et al. also did not reveal any evidence of the associations of rs2248374 and rs2549782 polymorphisms in the ERAP2 gene and susceptibility to AS. This is the only published meta-analysis of ERAP2 SNPs in AS including A total of six studies including 2774 AS patients and 4119 disease-free controls were eligible for this meta-analysis. Five studies reported rs2248374 polymorphism and three studies reported rs2549782 polymorphism. There were no statistically significant association with AS susceptibility: rs2248374, A vs. G, OR = 0.94, 95%CI 0.86–1.02, *P* = 0.14; rs2549782, T vs. G, OR = 1.03, 95%CI 0.95–1.12, *P* = 0.45 [28].

This research provides evidence of a statistically significant association between the rs2248374 genetic variant and variables related to occupation, drugs, smoking, and nonsteroidal anti-inflammatory drugs (NSAIDs). The findings of our study were corroborated by the research conducted by Bugaj et al., [29], which demonstrated a statistically significant association among rs2248374 and NSAIDs (*p* < 0.05). Furthermore, a strong correlation between rs2248374 and smoking was observed by Robinson et al., (*p* < 0.05) [24].

This research elucidated that a statistically significant disparity existed about rs2910686 concerning smoking, BASDAI, BASFI, and BASMI. The findings of our study were corroborated by Bugaj et al., [29], as they also observed significant relationships between the rs2910686 genetic variant and both the VAS and BASDAI scores.

This research provides evidence of a statistically significant disparity in the variables of rs2910686 concerning mSASSS, C.R.P, E.S.R, and Vitamin D.

In a similar vein, Bugaj et al., [29] discovered a noteworthy association between rs2910686, ESR, and CRP, with a statistical significance seen at a *p*-value of less than 0.05. Moreover, the study conducted by Robinson et al., [24] revealed a statistically significant correlation between rs2910686 and vitamin D levels (*p* < 0.05).

by univariate logistic regression, the rs2248374 SNP G allele and the rs2910686 SNP C allele were significantly upregulated in the AS patient group compared to the healthy control group with OR (LL – UL 95% C.I) of 1.198 (0.809–1.774) and 2.660 (1.741–4.064), respectively. Our findings are in consistent with the findings reported by Cortes et al. [25], who stated that upon doing a controlled analysis of the ERAP1 interaction with the ERAP2 polymorphism, a significant connection was identified between ERAP2 SNPs and AS instances. Specifically, the rs2910686 SNP exhibited an OR of 1.19 and a *p*-value of 0.213 × 10 − 2. Also, the study conducted by Ebrazeh et al. [30], revealed a statistical connection between the rs2910686 SNPs and the risk of AS in HLAB27 positive Iranian population.

## Limitations of the study

The present research is subject to some limitations, with one notable constraint being the relatively small sample size, which has the potential to impact the validity and generalizability of our findings. Therefore, it is essential to conduct more research with more extensive sample sizes to demonstrate the validity and reliability of our findings. The anticipated focus of future study should be on the therapeutic impacts of these sensitive biomarkers, rather than their diagnostic implications.

## Conclusion

The ERAP2 gene SNPs exhibited a significant correlation with AS susceptibility and clinical characteristics. However, it was shown that these SNPs exhibited significant associations with the serum levels of the inflammatory markers including ESR and CRP. Additional investigations of this gene, especially in haplotype analysis, are required to provide a more comprehensive understanding of the genuine contribution of ERAP2 SNPs to the development and progression of AS.

## Data Availability

The article incorporates the data used to substantiate the outcomes of this investigation.
